# Two for the Price of One: A Case of Methicillin-Resistant Staphylococcus aureus (MRSA) Brain Abscess With Atypical Lymphoplasmacytic Infiltrate With Underlying Clonal Lymphoproliferative Process in a Patient Infected With HIV

**DOI:** 10.7759/cureus.33325

**Published:** 2023-01-03

**Authors:** Frailyn J Nunez, Shorabh Sharma, Michelle Dahdouh

**Affiliations:** 1 Internal Medicine, SBH (St. Barnabas Hospital) Health System, Bronx, USA; 2 Infectious Disease, SBH (St. Barnabas Hospital) Health System, Bronx, USA

**Keywords:** clonal lymphoproliferative process, lymphoplasmacytic infiltrates, mrsa brain abscess, ring-enhancing lesions, hiv

## Abstract

Multiple etiologies can coexist and trigger CNS symptoms in individuals infected with HIV. This article reviews a case of a cerebellar mass in an HIV patient who, on biopsy, grew methicillin-resistant *Staphylococcus aureus* (MRSA) and by pathology, showed an atypical lymphoplasmacytic infiltrate concerning a clonal lymphoproliferative process, which may be a precursor of CNS lymphoma. The patient, a 36-year-old male with multiple comorbidities including HIV Infection, presented to the hospital for evaluation of a one-week course of headache and photophobia. Remarkable physical examination findings included dilated pupils and anisocoria. Initial CT brain imaging revealed vasogenic edema seen throughout the left cerebellar hemisphere provoking mass effect on the fourth ventricle and pontocerebellar cistern resulting in mild hydrocephalus, new findings compared to prior. MRI brain displayed a T1 isointense, T2 hypointense ring-enhancing lesion in the left cerebellar hemisphere, with restricted diffusion, and surrounding vasogenic edema resulting in mass effect over the fourth ventricle, left cerebellar peduncle, and pontocerebellar cistern causing mild hydrocephalus. The patient underwent left suboccipital craniotomy with evacuation of the cerebellar lesion by neurosurgery. Tissue cultures grew MRSA. Pathology was sent to New York-Presbyterian Columbia University Irving Medical Center due to the presence of atypical lymphoplasmacytic infiltrates. The final diagnosis was polyclonal B-cell population in the sample; however, prominent peaks were also seen above the polyclonal background, possibly representing a clonal proliferation. Therefore, the lymphoplasmacytic infiltrates remained atypical and the possibility of the underlying clonal lymphoproliferative process could not be entirely ruled out.

## Introduction

Changes in mental status, abnormal neurological examination findings, and/or lesions in brain imaging are frequently encountered in patients infected with human immunodeficiency virus (HIV). Ranges in CD4+ cell count have been associated with the development of changes in cognition and can be used as guidance for the evaluation of such a clinical presentation. Multiple etiologies can coexist and trigger central nervous system (CNS) symptoms in individuals infected with HIV. This article reviews a case of a cerebellar mass in an HIV patient who, on biopsy, grew methicillin-resistant *Staphylococcus aureus* (MRSA), and by pathology showed an atypical lymphoplasmacytic infiltrate concerning for a clonal lymphoproliferative process, which might have been a precursor of CNS lymphoma.

## Case presentation

The patient is a 36-year-old male with a history of recent intravenous (IV) crystal meth use, schizophrenia, chronic Hepatitis B infection, and HIV Infection compliant with his antiretroviral therapy (ART) medication regimen, unsure timing since starting medication. The patient affirmed undetectable viral load, with unknown CD4+ cell count, and a previous history of syphilis treated multiple times with penicillin with rapid plasma reagin (RPR) improved from 1:128 to 1:16, and presented to the hospital for evaluation of a one-week course of headache and photophobia that began before leaving against medical advice from another healthcare institution where he was evaluated for the same. Brain imaging at that time was significant for the presence of vasogenic edema of the left cerebellar hemisphere provoking mass effect on the fourth ventricle. 

**Figure 1:**
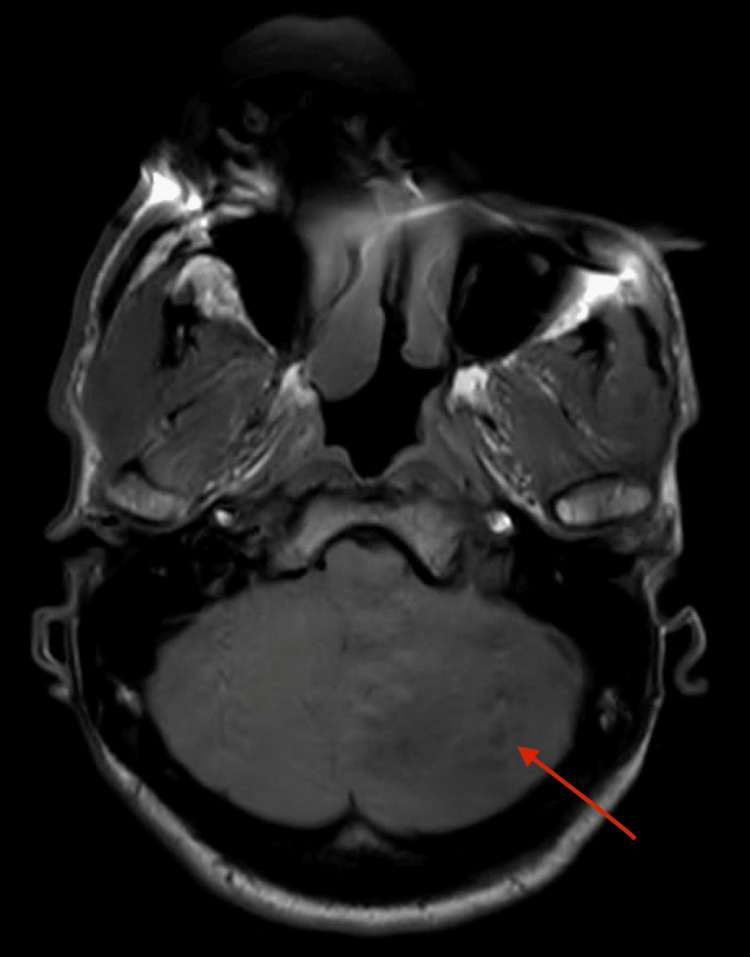
T1 series of magnetic resonance imaging (MRI)

**Figure 2:**
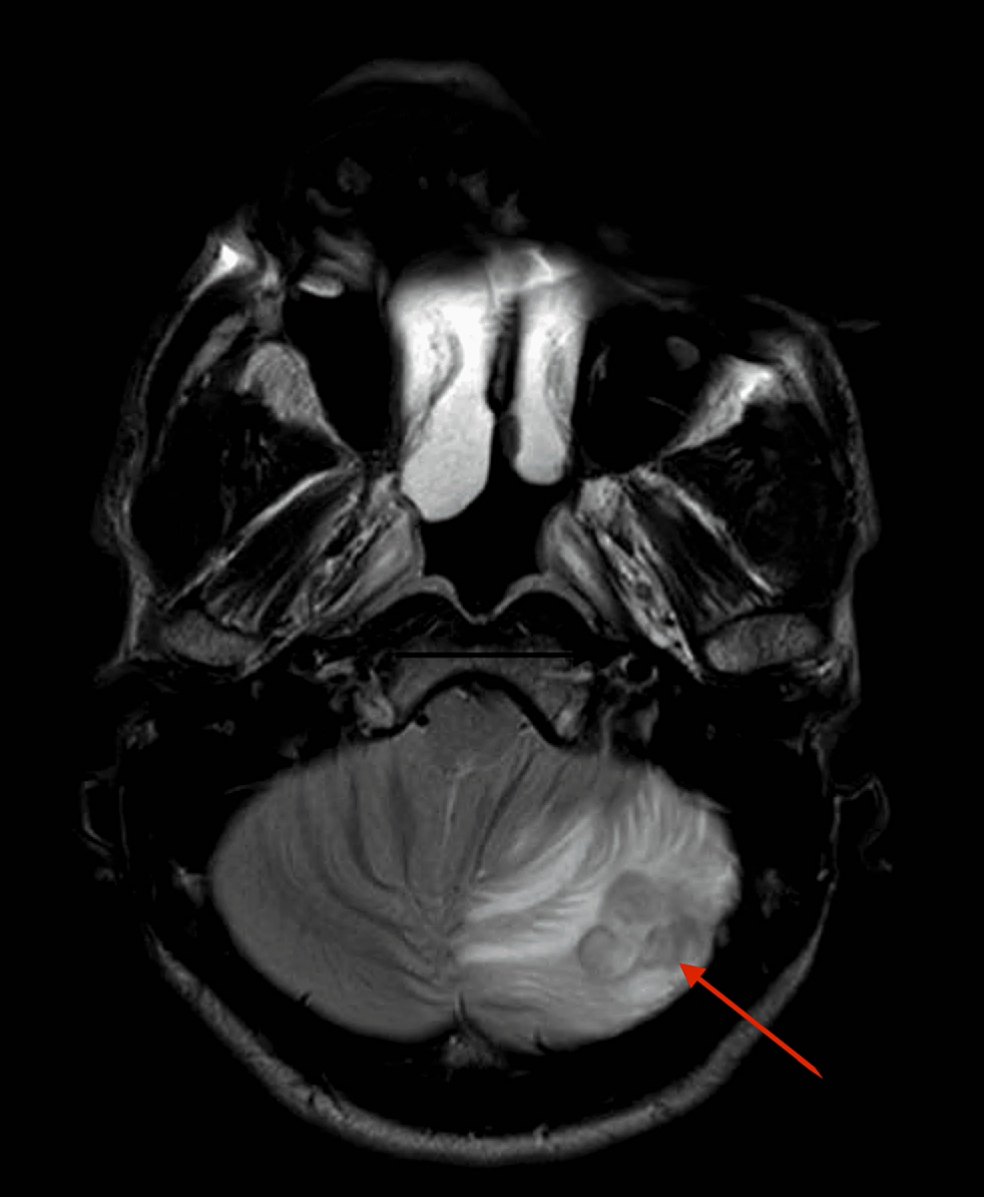
T2 series of magnetic resonance imaging (MRI)

Remarkable clinical examination findings included dilated pupils and anisocoria. Initial CT brain imaging evaluation revealed vasogenic edema seen throughout the left cerebellar hemisphere provoking mass effect on the fourth ventricle and pontocerebellar cistern resulting in mild hydrocephalus. These were new findings compared to the prior concerning for an active infectious condition versus malignancy. Magnetic resonance imaging (MRI) of his brain displayed a T1 isointense (Figure 1), T2 hypointense (Figure 2) ring-enhancing lesion in the left cerebellar hemisphere, with restricted diffusion, and surrounding vasogenic edema resulting in mass effect over the fourth ventricle, left cerebellar peduncle, and pontocerebellar cistern causing mild hydrocephalus. A cerebellar abscess in the context of recent IV drug use was in the differential, and the patient was commenced on broad-spectrum antibiotics after obtaining blood cultures. Other differentials included metastatic tumors and hemangioblastoma. The infectious disease department was consulted, and trimethoprim-sulfamethoxazole combination was also started since pyrimethamine was not available to cover for possible CNS toxoplasmosis.

Computer tomography (CT) scanning of the chest, abdomen, and pelvis was negative for malignancy. The patient’s CD4+ cell count came back as 211 cells/mm^3^, with an undetectable viral load. The rest of the viral panel work-up including serum herpes simplex virus-1 and 2, cytomegalovirus, and Epstein-Barr virus were negative by polymerase chain reaction (PCR) testing. Serum toxoplasmosis antibody, cryptococcal antigen, QuantiFERON (QIAGEN N.V., Venlo, The Netherlands), and blood cultures resulted negative. The RPR titer had appropriately decreased to 1:8. 

**Figure 3:**
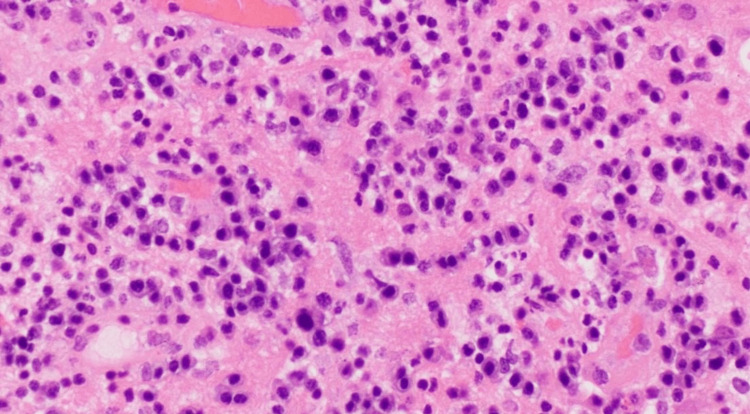
Brain biopsy demonstrating lymphoplasmacytic infiltrate

The patient underwent left sub-occipital craniotomy with evacuation of the left cerebellar lesion by neurosurgery. Tissue cultures grew MRSA, and fungal and acid-fast bacillus cultures were negative. Pathology was sent to New York-Presbyterian Columbia University Irving Medical Center due to atypical lymphoplasmacytic infiltrates seen in pathology (Figure 3). The final diagnosis was polyclonal B-cell population in the sample. However, prominent peaks were also seen above the polyclonal background, which could possibly represent a clonal proliferation; therefore, the lymphoplasmacytic infiltrates remain atypical, and the possibility of underlying clonal lymphoproliferative process cannot be entirely ruled out. No definitive microorganisms were seen with Grocott’s methenamine silver (GMS) special stains and with immunostainings for toxoplasma, mycobacteria, and John Cunningham (JC) virus (SV40). No definitive Gram-positive bacteria are seen in Gram-special stains. No definitive evidence for microorganisms is identified in spirochete staining. The echocardiogram showed normal left ventricular ejection fraction, with normal left ventricular wall motion. No vegetation was noted. The patient was to have a trans-esophageal echocardiogram, and antibiotics were de-escalated to vancomycin with an improving neurological exam; however, he signed out against medical advice.

## Discussion

Cerebral abscesses are considered pus-filled pockets of material with areas of necrosis within the brain parenchyma, usually resulting from a localized trauma or an infectious process. Based on recent studies, the incidence of brain abscesses is approximately 8% of intracranial masses, and its prevalence is even higher in patients with acquired immunodeficiency syndrome (AIDS) [1-3].

Brain abscesses go through different stages in their development; the first two weeks are characterized by the development of inflammation and focal cerebritis, followed in the next three to four weeks by the development of necrosis and liquefaction, covered by a capsule of granulation tissue, collagen deposition and surrounding edema [1-3].

The two leading diagnoses associated with mass effect to the brain in developed countries are toxoplasma encephalitis, followed by primary CNS lymphoma. In developing countries where tuberculosis and parasitic infections are highly endemic, CNS tuberculoma and neurocysticercosis are to be considered in the differential depending on the host’s immune system condition. Further evaluation for opportunistic infections may be guided by the characteristics of the CNS lesion, the presence or lack of mass effect, the size, and its location.

Brain abscesses caused by *Staphylococcus* and *Streptococcus* species, *Mycobacterium avium* complex, *Salmonella*, *Nocardia*, *Listeria*, *Rhodococcus*, cryptococcomas, and syphilitic gummas should be considered in the differential of CNS lesions in immunocompromised patients [4,5]. Our patient had a history of syphilis that was treated and had an appropriate decrease in RPR titers, as well as a negative spirochete stain on brain biopsy. His CD4+ cell count was just over 200 cells/mm^3^, placing him at low epidemiological and immunological risk for toxoplasmosis and primary CNS lymphoma; however, cases have been reported in patients with a CD4 count over 200 cells/mm^3^ [6]. Immune Reconstitution inflammatory syndrome (IRIS) may play a part in these cases.

Brain abscesses pose a threat, and may increase morbidity and mortality, due to the increased risk of them progressing to meningitis, ventriculitis, and even herniation. Therefore, medical management, or a combination with a surgical approach, including aspiration and excision of the lesion (s) is commonly considered for cure.

Primary CNS lymphoma, on the other hand, is an Epstein-Barr Virus (EBV)-related pathology, likely occurring due to ineffective immunoregulation in patients with HIV. Most lesions are usually supratentorial, can be solitary or multifocal, and typically feature isodensity or hyperdensity to gray matter, with surrounded hypodensity due to cerebral edema on CT brain, and central necrosis and hemorrhage on MRI. If glucocorticoids have not been administered to the patient, almost all demonstrate prominent enhancement upon intravenous contrast injection. First-line therapy includes a combination of ART and high-dose methotrexate (MTX) in patients who can tolerate it; however, the prognosis is guarded in cases when these lesions are found in an advanced stage, and in patients who cannot tolerate high-dose methotrexate due to renal insufficiency.

## Conclusions

Primary CNS lymphoma is still a relatively rare primary brain tumor, even with the recent surge of AIDS cases worldwide. Opportunistic CNS infections are an even more common etiology of brain lesions in patients with HIV. For instance, toxoplasmosis is by far the most frequent parenchymal infection found in AIDS patients. This case report highlights the need for clinicians to be mindful that other rare entities such as brain abscesses can coexist in selected groups at risk, and that early intervention is paramount to avoid further morbidity and mortality. Our patient was diagnosed with MRSA brain abscess through culture evaluation and by pathology possible underlying clonal lymphoproliferative process, which may be a precursor of CNS lymphoma. The presence of HIV can cause diagnostic dilemmas and require an open mind in diagnostic possibilities. 
